# Improved camouflage through ontogenetic colour change confers reduced detection risk in shore crabs

**DOI:** 10.1111/1365-2435.13280

**Published:** 2019-01-24

**Authors:** Ossi Nokelainen, Ruth Maynes, Sara Mynott, Natasha Price, Martin Stevens

**Affiliations:** ^1^ Centre for Ecology and Conservation, College of Life and Environmental Science University of Exeter Penryn UK; ^2^ Department of Biological and Environmental Science University of Jyväskylä Jyväskylä Finland

**Keywords:** background matching, *Carcinus maenas*, disruptive coloration, ontogenetic colour change, phenotypic plasticity, predation, vision model

## Abstract

1. Animals from many taxa, from snakes and crabs to caterpillars and lobsters, change appearance with age, but the reasons why this occurs are rarely tested.
2. We show the importance that ontogenetic changes in coloration have on the camouflage of the green shore crabs (*Carcinus maenas*), known for their remarkable phenotypic variation and plasticity in colour and pattern.
3. In controlled conditions, we reared juvenile crabs of two shades, pale or dark, on two background types simulating different habitats for 10 weeks.
4. In contrast to expectations for reversible colour change, crabs did not tune their background match to specific microhabitats, but instead, and regardless of treatment, all developed a uniform dark green phenotype. This parallels changes in shore crab appearance with age observed in the field.
5. Next, we undertook a citizen science experiment at the Natural History Museum London, where human subjects (“predators”) searched for crabs representing natural colour variation from different habitats, simulating predator vision.
6. In concert, crabs were not hardest to find against their original habitat, but instead, the dark green phenotype was hardest to detect against all backgrounds.
7. The evolution of camouflage can be better understood by acknowledging that the optimal phenotype to hide from predators may change over the life history of many animals, including the utilization of a generalist camouflage strategy.

A plain language summary is available for this article.

## 1 INTRODUCTION

Camouflage is key to survival in numerous organisms. It is a widespread anti‐predator strategy; whereby, organisms avoid detection or recognition by resembling the general background or specific objects within the habitat (Cott, 1940; Nokelainen & Stevens, 2016; Ruxton, Sherratt, & Speed, 2004; Stevens & Merilaita, 2011). The efficacy of camouflage is linked to the similarity of individuals with features of the visual environment (Troscianko, Wilson‐Aggarwal, Spottiswoode, & Stevens, 2016), and therefore, generally a given phenotype should be effective in hiding individuals in some environments but not in others (Ruxton et al., 2004; Stevens & Merilaita, 2009). Importantly, camouflage is often not static because many animals can change appearance over time during their life span, either through reversible plastic changes or via ontogenetic changes (Duarte, Flores, & Stevens, 2017; Stuart‐Fox & Moussalli, 2009). Yet, the mechanisms and implications of ontogenetic colour change for survival remain significantly unexplored. This is in part because quantifying long‐term changes in camouflage while controlling for different backgrounds is challenging, and because the majority of work to date has focussed on short‐term plastic and/or reversible change.

Colour change is commonplace in nature, occurring both in invertebrates (e.g., insects, crustaceans and molluscs; Bedini, 2002; Barbosa et al., 2008; Eacock, Rowland, Edmonds, & Saccheri, 2017; Valkonen et al., 2014) and in vertebrates (e.g., fish, amphibians, reptiles and mammals; Akkaynak, Siemann, Barbosa, & Mäthger, 2017; Booth, 1990; Kang, Kim, & Jang, 2016). For instance, many crustaceans can change their appearance depending on the habitat for increased similarity with the visual environment over a period of hours and days (Brown & Sandeen, 1948; Powell, 1964; Rao, Fingerman, & Bartell, 1967; Stevens, Lown, & Wood, 2014a; Stevens, Rong, & Todd., 2013). Similar changes for camouflage tuning over days and weeks occur both within and between moults in other groups, such as grasshoppers (Burtt, 1951; Edelaar, Baños‐Villalba, Escudero, & Rodríguez‐Bernal, 2017; Peralta‐Rincon, Escudero, & Edelaar, 2017) and caterpillars (Eacock et al., 2017). Not only can individuals change their coloration over multiple time‐scales to facilitate camouflage, but many also undergo changes in appearance as a result of ontogeny (Duarte et al., 2017; Iampietro, 1999; Jensen & Egnotovich, 2015; Reid, Abello, Kaiser, & Warman, 1997; Stevens, 2016; Styrishave, Rewitz, & Andersen, 2004; Todd, Qiu, & Chong, 2009). For example, racer snakes become more uniform in coloration with age, a change that seems to be linked to behaviour and anti‐predator strategies (Creer, 2005). In certain tropical pythons, juveniles can be variable in coloration but switch to a green appearance in adulthood, seemingly to provide camouflage from predators in different habitats (Wilson, Heinsohn, & Endler, 2007). Furthermore, many crabs undergo ontogenetic colour changes and their phenotypic diversity has been suggested to mirror habitat‐specific camouflage against visually guided predators (Palma & Steneck, 2001; Stevens, Lown, & Wood, 2014b; Todd, Briers, Ladle, & Middleton, 2006; Todd, Oh, Loke, & Ladle, 2012). These may link to size‐related habitat changes and have fitness consequences as growth and survival may both be improved in the new habitat (Hultgren & Mittelstaed, 2015; Hultgren & Stachowicz, 2010, 2011).

Many marine crustaceans are extremely variable in appearance among individuals in early life, with intraspecific diversity in colour and patterning declining with age (Anderson, Spadaro, Baeza, & Behringer, 2013; Booth, 1990; Carvalho‐Batista et al., 2015; Duarte et al., 2017; Krause‐Nehring, Matthias Starck, & Richard Palmer, 2010; Palma & Steneck, 2001; Todd et al., 2009). However, the reasons for such ontogenetic changes have seldom been experimentally explored and remain somewhat mysterious, but may reflect a reduction in predator risk as individuals grow larger and become more defended (thus have a reduced need for camouflage), or a switch to different habitat types with age (Hultgren & Stachowicz, 2010; Todd, 2009; Wilson et al., 2007). As these ideas have rarely been properly tested, it remains unknown what effect development has on camouflage efficacy and how ontogenetic changes interact with reversible plastic changes. Previous work in snakes has shown links between ontogenetic colour change, camouflage (modelled to predator vision) and behaviour (Wilson et al., 2007), but has not directly measured how detection or survival is affected by such colour changes (but see Hultgren & Mittelstaed, 2015). In addition, few, if any, studies have performed experiments to determine how ontogenetic changes arise and interact with plastic reversible changes. Hence, there is a lack of empirical studies addressing whether developmental changes in coloration actually link to reduced attack risk by predators and have the potential to be adaptive.

Here, we examined how ontogenetic and plastic changes in appearance influence camouflage efficacy in the green shore crab (*Carcinus maenas*). Adult shore crabs have shown to be more uniform in colour and pattern than juveniles (Hogarth, 1978; Stevens, 2016; Stevens et al., 2014a; Todd, Ladle, Briers, & Brunton, 2005), plausibly due to ontogenetic changes in coloration. In addition, juvenile shore crabs are capable of changing brightness (i.e., lightness) and colour (i.e., chromatic changes) over a period of hours (Powell, 1964; Stevens et al., 2014a), and over weeks, including through moulting to better match the background (Stevens, 2016). Such longer‐term changes are reversible, with crabs changing to dark colours on dark backgrounds and light colours on light backgrounds.

Our first aim was to study whether juvenile shore crabs adjust their coloration (i.e., both colour and pattern) over successive moults in order to increase their background resemblance to substrates representing different habitats. We conducted a 2×2 factorial common garden experiment, where we reared juvenile shore crabs of two initial shades (pale or dark) on two artificially created naturalistic background types (resembling rock pool or mudflat) for 10 weeks. We predicted that crabs would adopt a coloration that would improve their background matching (Iampietro, 1999; Stevens, 2016; Stevens et al., 2014a, 2013). Specifically, crabs growing on “rock pool” backgrounds should develop more contrasting and variable patterns, whereas crabs growing on “mudflat” background should develop greener colour and uniform patterning. Second, to evaluate the potential survival benefit associated with changes in coloration, we conducted a factorial predation experiment, using humans as model “predators” (Bond & Kamil, 2002; Sherratt & Beatty, 2003; Todd, 2009). We used a citizen science game, based at the Natural History Museum in London, UK, where subjects search for crabs representing natural colour variation on touch screen and detection times were measured (similar to a recent study on camouflage in birds; Troscianko, Wilson‐Aggarwal, Griffiths, Spottiswoode, & Stevens, 2017). Crab and background images originated from nine locations from three habitat types (rock pool, mudflat and mussel bed), with crabs of randomized sizes presented against each background type with the display simulating a trichromatic (e.g., human) or dichromatic (e.g., fish) visual system (see Section 2). We predicted that crabs would be harder to find against visually more complex backgrounds (Bond & Kamil, 2002; Karpestam, Merilaita, & Forsman, 2014; Punzalan, Rodd, & Hughes, 2005) and that crabs would be harder to find against the background type from where they originated, assuming that they possess background‐specific camouflage (Moran, 1992; Stevens et al., 2015; Todd et al., 2006, 2012). We also tested for differences in detection by di‐/trichromatic vision systems (Troscianko et al., 2017). To our knowledge, our study is the first direct demonstration that ontogeny drives a generalist camouflage strategy linked to age in a manner that promotes survival.

## 2 MATERIALS AND METHODS

### 2.1 Colour change experiment

The experiment was conducted at the University of Exeter, Penryn Campus, Cornwall, between February and May 2016. Individual crabs used for the common garden experiment were collected from the Gyllyngvase beach (coordinates in decimal degrees: 50.141888, −5.063811), Cornwall, UK, during February 2016. Shore crabs are located in a wide range of habitat and substrate types around the shore, each with different appearances, including estuaries, mudflats, sandy beaches, shingle, pebbles, mussel beds and rocky coastline (Brian, Fernandes, Ladle, & Todd, 2006; Crothers, 1968; Edwards, 1958; Stevens et al., 2014b; Todd et al., 2006, 2012). The collection methods largely follow established protocols (Nokelainen, Hubbard, Lown, Wood, & Stevens, 2017; Stevens et al., 2014b). Briefly, the crabs were collected by hand during low tide alongside the beach from approximately 50 m length, and thus, our sampling included crabs from different substrates. Crabs were transported from nearby tidal pools into the laboratory immediately after capture. Crabs entering the experiment were all of similar size, approximately 15 mm carapace width. After collection, crabs were photographed and divided into experimental groups based on their carapace lightness in a randomized block design (i.e., crabs with contrasting lightness were equally represented in treatment groups, see further). Crabs were photographed once a week and after moulting. Shore crabs are not a protected species, and all work was conducted under approval from the University of Exeter Biosciences Ethics Committee (applications 2013/75 and 2014/556). The field locations are publicly accessible; no further permits were needed.

**Figure 1:**
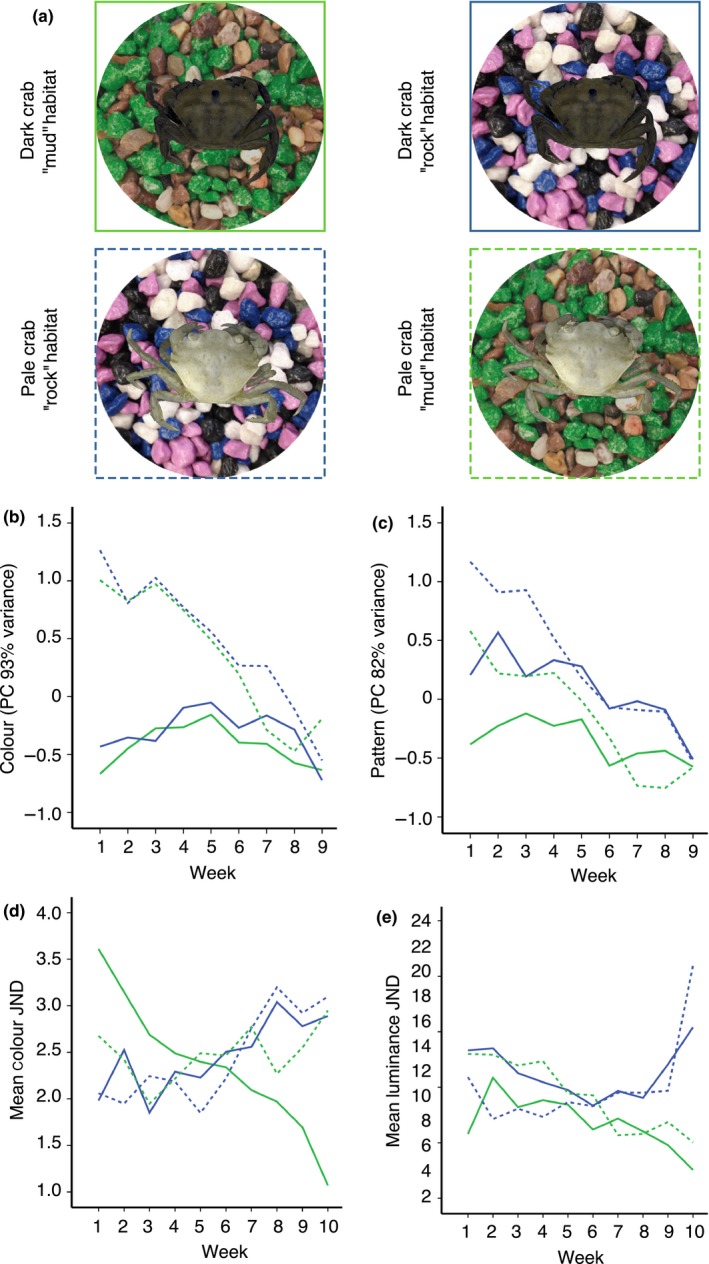
The long‐term development of background matching of *Carcinus maenas* for approximately 10 weeks of rearing under controlled conditions. A 2×2 factorial design was used utilizing two initial crab colour types and two rearing backgrounds in a common garden experiment (a). Two artificial background types, mudflat and rock pool, were both constructed using aquarium gravel. The crabs representing two initial shade types, dark and pale, were reared on these background types, and changes in their carapace coloration were recorded. Lines around the crabs represent treatment group legends in the panels (b–e). Solid green: dark‐shaded crabs on mud background; Solid blue: dark‐shaded crabs on rock pool background; Dashed green: pale‐shaded crabs on mud background; and Dashed blue: pale‐shaded crabs on rock pool background. The change in colour (b) and pattern (c) principal components obtained from normalized camera responses. The effect of colour change to chromatic (d) and luminance (e) background match (modelled through predatory fish vision, JNDs, just noticeable differences)

First, we study whether juvenile shore crabs adjust their appearance (i.e., including both colour and pattern) within and over successive moults in order to increase their resemblance to heterogeneous substrates (unlike our previous work, which has tended to focus on more simplified uniform backgrounds; Stevens et al., 2014a, Stevens, 2016). Experimental animals were divided into four treatment groups using a 2×2 factorial set‐up with crabs of two shades (pale, dark) on two naturalistic background types (i.e., rock pool and mudflat—Figure 1). Carapace brightness was used to divide crabs in two distinct groups. Group discreteness was further validated based on the camera‐obtained spectral data (see below; ANOVA for carapace brightness between dark and pale treatment groups, *N* = 60, *F* = 34.15, *df* = 1, *p* < 0.001). Beginning with two unambiguous groups allowed us to control for the extensive phenotypic variation of juvenile crabs.

We chose background types in which to rear crabs that represent two common natural extremes: relatively homogeneous mudflat and more heterogeneous rock pool backgrounds. We replicated these backgrounds using standard aquarium gravel (UNIPAC) after subjective evaluation of their general properties of colour and pattern from photographs. “Mudflat” background was a mixture of brown and green (i.e., representing brown mud and green algae) aquarium gravel (1:1 ratio), whereas “rock pool” background was a mixture of black, grey, white and purple aquarium gravel (with equal ratios). We deliberately chose not to use actual natural substrates as this may contain chemical cues of predators or other stimuli that may influence crab development and that may also differ in texture/size as well as colour pattern, thereby hindering full control over the experiment. Using artificial gravel also enabled greater standardization of background samples among individuals. We compared the match of our artificial backgrounds to natural ones using calibrated photographic data (see below). Similarity of the backgrounds in a trichromatic RGB colour space was calculated based on reflectance data for brightness (i.e., average reflectance across all colour channels; R+G+B/3) and hue (i.e., red divided by blue channel). Artificial backgrounds represented similar albeit not perfectly matching natural variation of colourful tidal environments (Supporting Information Figure S1). In particular, the artificial backgrounds most effectively matched the brightness of their natural counterparts. In nature, rock pools harbour a great range of chromatic variability, both within and among patches, including pink‐coloured elements such as red encrusting coralline algae and also have blue‐coloured elements such as mussels. Mudflats instead are characterized by brown tones of wet soil and gravel and get mixed by green, brown and red algae. Therefore, although our artificial substrates are not a perfect match to the natural substrates, they are broadly representative, and crucially, the appearance of the mudflat and rock pool treatments is very different.

Altogether, we reared 60 crabs (17 in “dark‐mud” treatment, 16 in “dark‐rock” treatment, 13 in “pale‐mud” treatment and 14 in “pale‐rock” treatment) in customized aquarium tanks (90 × 45 cm in area) for 10 weeks. Each tank was divided into 24 similar sections (11 × 15 cm). The section walls were glued using adhesive silicon glue, and walls contained a mesh‐covered hole ensuring water circulation through the system. Tanks were filled with dechlorinated tap water mixed with artificial sea salt (Aquarium Systems Instant Ocean Salt; Swell UK Ltd., UK) to simulate natural seawater, which was tested with a refractometer (D&D's Refractometer; Swell UK Ltd.) to ensure salinity of 30 ppt. The water was passed through a filtration system (Eheim classic 350; EHEIM GmBH & Co. KG, Deizisau, Germany) and cooler (D&D DC300 aquarium cooler 300w cooling power; Swell UK Ltd.), keeping the water both clean and at a constant temperature. Temperature was set to 16°C to mimic local sea temperature at the time of collection. Two sections were not used to accommodate crabs, but instead housed the inputs and outputs of the filtration system to allow for maximum water flow through each section of the tank. An air stone (Aquarline High Output Air Compressor, 2,880 L/hr) was accompanied with the filter output section to allow as much oxygen to flow through the tank as possible. We used two daylight lamps and one near UV lamp (GroBeam 600 Ultima and AquaBeam 600 Ultima MW; Tropical Marine Centre UK) to simulate natural light conditions, which were controlled by a timer to establish a constant light cycle (12:12 L/D‐cycle). Crabs were fed daily with standard marine crustacean aquarium food. Water was changed, filters checked and tanks cleaned weekly to maintain living conditions of crabs. Some crabs did not survive through 10‐week experiment. However, mortality was not significantly different with regards to background type or crab initial shade, nor there was difference in moulting rates between the treatments.

### 2.2 Photography and vision modelling

Photography, initial image calibration and analysis broadly followed previously used methods (Stevens et al., 2014a). Full details are given in Supporting Information Table S1). Briefly, imaging was undertaken with a Samsung NX1000 digital camera converted to full spectrum with no quartz filter to enable UV sensitivity, and fitted with a Nikon EL 80‐mm lens. For the human visible photographs, we placed a UV and infrared (IR) blocking filter in front of the lens, which transmits wavelengths only between 400 and 680 nm (Baader UV/IR Cut Filter). For the UV images, a UV pass and IR blocking filter was used (Baader U filter), which transmits between 320 and 380 nm. Grey reflectance standards, which reflect light equally at 7% and 93% between 300 and 750 nm, were used.

For each image, we measured the entire dorsal side of the crab carapace to obtain colour and pattern information. We analysed the data both with normalized camera responses and with fish vision modelled data (see below). For reflectance data (i.e., colour), we used normalized camera responses of brightness, red, green, blue and UV channel. The pattern analysis technique (a “granularity” analysis) involved decomposing an image into a series of different spatial frequencies (“granularity bands”) using Fourier analysis and band‐pass filtering, followed by determining the relative contribution of different marking sizes to the overall pattern (Barbosa et al., 2008; Hanlon et al., 2009; Stoddard & Stevens, 2010). For the pattern data (see further details in Supporting Information Table S1), we used maximum power (i.e., pattern dominance—the energy at the spatial frequency with the highest pixel energy), proportional power (i.e., pattern diversity—maximum or peak energy value divided by the summed energy), total power (i.e., overall contrast or amplitude—the energy summed across all scales) and mean power (i.e., average contrast across the spectrum). Pattern analysis was conducted in custom files for ImageJ (Troscianko & Stevens, 2015).

To examine the level of background match, we calculated how changes in the crab carapace influenced their level of match to the experimental backgrounds. To do so, we used a receptor noise‐limited visual discrimination model (Vorobyev, Osorio, Bennett, Marshall, & Cuthill, 1998), which is based on differences in colour or luminance based on photon catch values. For calculations, all crabs were photographed weekly over the course of the experiment. Also, the backgrounds (i.e., aquarium gravel mixtures from the slots individual crabs were kept on) were photographed. Thus, different metrics (see below) were calculated between crab carapace and the very background each crab was reared on matching the size of the entire slot (c. 10 cm in diameter). We used a fish vision model based on the longwave (LW) and shortwave (SW) visual sensitivity of the pollack (*Pollachius pollachius*) (Shand, Partridge, Acher, Potts, & Lythgoe, 1988). A Weber fraction value of 0.05 was used for the most abundant cone type with receptor cone ratios of SW 168 and LW 339 (Govardovskii, Fyhrquist, Reuter, Kuzmin, & Donner, 2000). The receptor noise model yields values in “just noticeable differences” (JNDs); whereby, differences between 1 and 3 are interpreted that two stimuli are unlikely to be discriminated by an observer (and hence indicate a good background match). Larger values than this are increasingly likely to be discriminable, whereas values lower than this (<1 JND) should be virtually indistinguishable (Kelber, Vorobyev, & Osorio, 2003; Olsson, Lind, & Kelber, 2015; Siddiqi, Cronin, Loew, Vorobyev, & Summers, 2004). Caution must be used in interpretation of JNDs, because the method is sensitive to estimates of receptor noise, light conditions and animal cognition. As such, we follow past work and use a slightly broader region of uncertainty in discrimination thresholds (1–3 JNDs), but ultimately, the key consideration is that smaller JND values should equate to better camouflage match.

### 2.3 Visual predation computer detection experiment

To test camouflage efficacy of different crab phenotypes in varied backgrounds, we made a predation game where human participants searched for crabs of various sizes presented on a touch screen. Our main questions were as follows: Does the visual complexity of the background make it harder to find the prey, and are crabs hardest to find against their local habitat type (i.e., consistent with a background‐specific camouflage hypothesis)?

To obtain crab and background images for the game, we sampled crabs from nine locations around Cornwall in the southwest UK and photographed them. These intertidal sites represent backgrounds of different visual complexity (with higher complexity involving substrates of many textures, contrasts, colours, shapes and different‐sized granules). Here, rock pools represent subjectively the most visually complex (a–c), mussel beds medium (d–f) and mudflats the simplest (g–i) sites. Sites were as follows: (a) Falmouth (all coordinates in decimal degrees, 50.141888, −5.063811), on the south coast, comprising a stretch of shoreline collectively encompassing Castle and Gyllyngvase beaches. Sites hold rock pools with rocky crevices with stony or gravel substrates in the pools and, lower down on the shore, increasing abundance of seaweed. (b) Summers beach at St. Mawes (50.157095, −5.017370), on the south coast comprising rock pools, gravel and some low seaweed cover adjacent to a pebbled beach. (c) Flushing (50.162191, −5.066843), on the south coast comprising rock pools, gravel and seaweed cover. (d) Godrevy Point (50.249499, −5.320966), on the north coast, which primarily consists of exposed rocky outcrops with mussel beds. (e) Polzeath (50.576169, −4.920206), on the north coast of Cornwall, comprising mostly mussel bed cover adjacent to a beach. (f) Mawgan‐Porth (50.466705, −5.041101), on the north coast of Cornwall, comprising mostly mussel bed cover and pools adjacent to a beach. (g) Helford Passage (50.098763, −5.132556), an estuarine location on the south coast has a large mudflat area as well as tiered craggy rock pools. (h) Penryn (50.166956, −5.082634), mostly mudflats with a covering of green algae. (i) Hayle (50.188010, −5.428120), on the north coast of Cornwall, an estuarine location has a large mudflat area.

For the game, crabs as well as the natural backgrounds from the field sites were photographed using the methods described above. Briefly, we used calibrated Samsung NX1000 equipped with Nikon EL‐80 mm Nikkor and Nikon D7000 camera with a 60‐mm Coastal Optics lens. The crabs were detached from the background using GIMP2 image manipulation software, and the background images were cropped to 16:9 aspect ratios for the touch screen game. Crabs were scaled into the same pixel/mm aspect ratio to show crabs against the background images in natural size with respect to the background scale. Due to the number of crab images needed, custom software was designed (called “autocrab”) to automate the process of background subtraction. This software allowed users to step through hundreds of images, automatically loading, thresholding and flood filling background areas, saving them with an appropriate transparency channel in the correct format and resolution needed for the game. This created usable crab images for 80% of the photographs very easily, with some additional cleaning up required for the rest using GIMP2 image manipulation software (https://zenodo.org/record/1101057). DOI for the source code: 10.5281/zenodo.1099634.

The experiment was a part of the Colour and Vision exhibition at the Natural History Museum of London (NHM), UK, during autumn 2016. It followed the same general design of a previous online citizen science detection experiment to find hidden birds (Troscianko et al., 2017). Naturally, humans are not prime predators of crabs, but using this technique we were able to test visual detection under standardized conditions (see Section 4). Participants were visitors to the exhibition that clicked on a screen to accept their participation in the game and the use of their data. However, the data presented here only used the data collected at NHM. We collected basic player information, including player age and whether they had played the game before, but no personal information, and participants were free to quit the game at any time. There were two versions of the game, comprising displays that broadly simulated the information to a dichromatic observer (e.g., dichromatic combined red and green layers; simulating fish vision) and trichromatic (e.g., human) observer (Troscianko et al., 2017). However, we did not find significant difference in how quickly people found the prey in these two versions of the game, and so, we do not focus on these versions here. Prior to playing, the participants were asked to give their age group (<10, 10–15, 16–35, 36–50, >50, in order to control for any age effects), to state whether they had played the game before (to control for the multiple attempts, here we used only first plays) and to choose whether they would like to play as a simulated dichromat (“fish,” pollack vision) or a trichromat (human) vision. Participants were informed to click on the crab in each image as soon as they saw them. When participants successfully clicked on the target, their capture time was recorded (to the closest millisecond). The location of the target was made random in each slide without touching the edges of the screen. Participants were given 30 s to find the target in each slide. If they found the crab on time, it was included as “hit.” If they failed to find the crab within time limit, their data were considered as “miss,” they were given a “time‐is‐up‐message,” and the target crab was highlighted on a screen after which the player could move onto the next slide. A total of 20 slides were presented in each game trial. Each person saw a set number of random slides per treatment combination (i.e., a randomized block design). At the end, mean capture time was displayed and a summary of results was shown.

To investigate colour and luminance discrimination values in the citizen science game, we also used the Vorobyev & Osorio (1998) receptor noise‐limited vision model. For this, we used colour and luminance contrasts based on human vision to predict crab camouflage to humans in the experiment. We used human longwave (LW), mediumwave (MW) and shortwave (SW) sensitivity data and Weber fractions after Hofer et al. 2005: LW 0.020, MW 0.028 and SW 0.066 with receptor cone ratios LW 0.629, MW 0.214, and SW 0.057 for the human vision chromatic contrast, and 0.1 for luminance contrast (based on the human achromatic channel of LW + MW). Unfortunately, we could not analyse the appearance of the crabs and images as displayed to participants in situ on screen that the NHM London provided for the exhibition. Thus, for detectability comparisons we used a subset of crabs presented against experimental backgrounds of each treatment group resulting in following comparisons in our 3x3 factorial set‐up: mudflat crab against mudflat (*n* = 99), mudflat crab against mussel bed (*n* = 110), mudflat crab against rock pool (*n* = 88), mussel bed crab against mudflat (*n* = 108), mussel bed crab against mussel bed (*n* = 99), mussel bed crab against rock pool (*n* = 96); rock pool crabs against mudflat (*n* = 108), rock pool crab against mussel bed (*n* = 120) and rock pool crabs against rock pool (*n* = 96). Note that here we have not analysed pattern match of crabs to each background, which requires a number of approaches, and visual detection will depend not just on colour and luminance match but also on pattern.

### 2.4 Statistical analyses

We used linear mixed‐effects analyses (LMER) to analyse developmental of background matching through ontogeny common garden data. For colour and pattern characterization, we first used principal component analysis. We did this in order to reduce data dimensionality, because we wanted to integrate all colour as well as pattern metrics into single dependent variables for the analyses. For reflectance data (colour), we used normalized camera responses of brightness, red, green, blue and UV, which yielded one component (PC_colour_) explaining 93% of the variance with an Eigenvalue 4.65. For pattern data, we used maximum power, proportional power, sum power and mean power, which yielded one component (PC_pattern_) explaining 82% of the variance with an eigenvalue 3.26. We also calculated colour and luminance JNDs (i.e., just noticeable differences using a fish vision model, see above).

To analyse colour change experiment data, PC_colour_, PC_pattern_, chromatic JND match and luminance JND match were used separately as dependent variables. Crab initial appearance, background, week and their interactions were set as fixed factors. Tank and crab ID were set as random factors. Similarly, we analysed the following additional colour and pattern metrics for the supplementary material: luminance, hue, pattern diversity, pattern contrast and marking size (see Supporting Information Tables S2 and S3). Model simplification here and on further analyses was conducted according to the lowest Akaike information criterion value when necessary to improve the model fit (i.e., to test whether removing term of interest does not significantly impair the model fit), although full models often held the best fit to the data. Results remained similar if a traditional maximum likelihood test to compare a full model with a simplified model without the combination of interest (i.e., using backward stepwise protocol with significant departures from chi‐square distribution) was applied.

To analyse computer‐based predation experiment data, we first tested whether finding crabs is more difficult against certain backgrounds using generalized linear mixed modelling. The success of finding the crab correctly on time (hit, miss) was set as a binomial dependent variable. Similarly, we ran another analysis using LMER where we used search time as a dependent variable. In both of these analyses, crab habitat, photo habitat, vision system (tri‐/di‐chromatic; this, however, was omitted from the final models) and their interactions were set as fixed factors. Crab size was set as a random covariate. Also, the game ID was set as a random factor to account for games with different players and settings. Similarly, we ran two LMER analyses to analyse crab detectability, using luminance and chromatic match (separately) as dependent variables and crab ID as random factor. All analyses were done with IBM SPSS Statistics (v22) and program R (3.2.1).

## 3 RESULTS

### 3.1 Developmental plasticity and colour change

**Figure 2:**
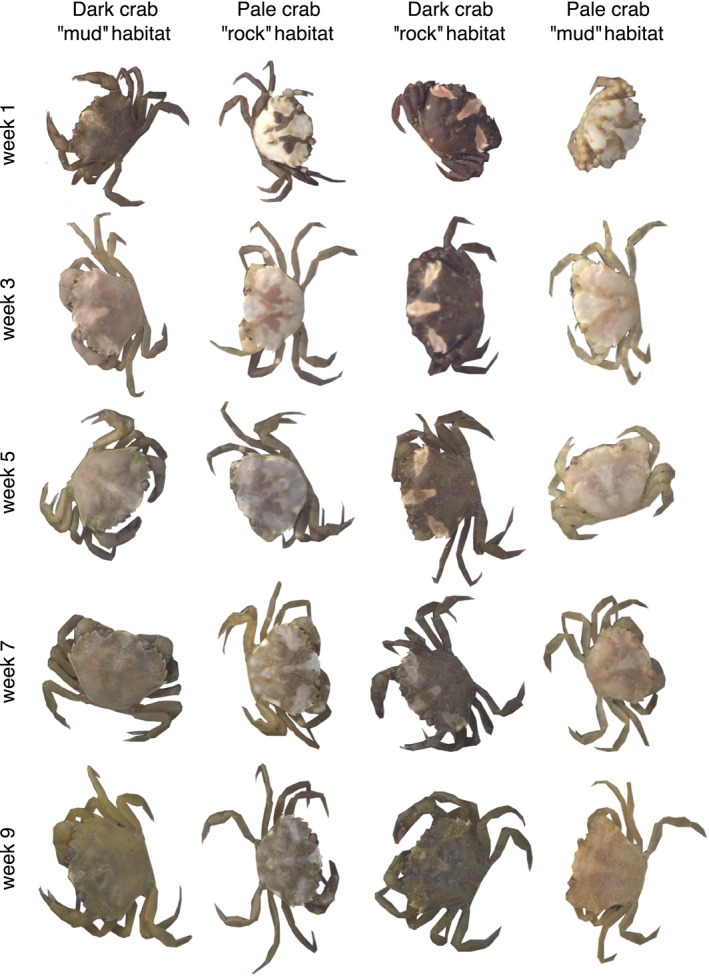
Ontogenetic changes in the green shore crab (*Carcinus maenas*). The figure illustrates that crabs converge on a similar phenotypic domain as a function of time. The crabs in columns are examples of individual crabs reared on different treatments, with the starting point at the top and end at the bottom. First column is a dark crab on mud background, second is a pale crab on rock background, third is a dark crab on rock background, and fourth is a pale crab on mud background. The rows show phenotypic change over time, here shown at start and then every second week. Figure is not to scale

We reared 60 crabs under common garden conditions for 10 weeks during which all individuals adopted a dark green/brown (i.e., “mudflat”) phenotype. The fact that crabs developed a darker carapace over time was indicated by decrease in luminance (i.e., lightness) and changes in reflectance values in all treatment groups (Table 1, Figure 1, Supporting Information Table S2). Crab colour (PC_colour_) was significantly associated with crab initial shade and time indicating that colour (i.e., relative contribution of normalized UV, SW, MW and LW wavelength bands) was different between treatment groups and that these changed over the course of experiment (Supporting Information Figure S2). This was markedly caused by colour shift to middle wavelengths over the course of time (i.e., becoming greener with respect to other colour channels). Crabs also went through developmental changes in terms of pattern diversity, contrast and marking size, with all metrics decreasing over time indicating shift to a more uniform carapace patterning (Figures 1 and 2, Supporting Information Table S3). Crab pattern (PC_pattern_) was associated by the interaction between week and shade, which was caused by darkened appearance of crabs over time being especially so in pale‐shaded crabs (Table 1).

**Table 1:** Linear mixed‐effects analyses (LMER) testing the developmental colour and pattern change of crabs as obtained from normalized camera responses

Subject	Estimate	*SE*	*df*	*t*‐Value	*p*
Crab colour (PC_colour_)					
(Intercept)^a^	0.09	0.21	1.8	0.43	0.708
Shade [pale]	0.87	0.20	38.3	4.17	<0.001
Time [week]	−0.10	0.01	437.9	−8.83	<0.001
Crab pattern (PC_pattern_)					
(Intercept)^a^	−0.09	0.29	2.2	−0.32	0.776
Background [rock pool]	0.67	0.24	65.0	2.75	0.007
Shade [pale]	0.81	0.24	64.9	3.29	0.001
Time [week]	−0.06	0.02	345.1	−3.15	0.001
Background × Week	−0.04	0.02	346.9	−1.77	0.076
Shade × Week	−0.10	0.02	346.5	−3.99	<0.001

Note

LMER predicts the colour and pattern responses in relation to crab original appearance (“shade”), rearing background type (“background”), time (“week”) and their interactions. Intercept includes rearing tank and crab ID as random variables.

a Intercept includes factor level: Background [mud] and Shade [dark].

Unexpectedly, we did not find evidence that crabs consistently improved background match to the specific backgrounds on which they were kept. Both luminance and chromatic camouflage match (as measured in discrimination values, JNDs, using a fish vision model) declined to a closer match on mud than rock background (Figure 1, Table 2), because of the dark green phenotype the crabs adopted. In both luminance and chromatic matching, there was a significant three‐way interaction among background, crab shade and time (Table 2). Background match of initially pale crabs became worse, whereas match of initially dark crabs became better over time, and crabs kept on “mud” background developed better match than crabs kept on “rock pool” background. However, only dark crabs on “mud” background were consistently able to improve the background match. The closest luminance match was achieved by dark crabs on “mud” background (${\overset{¯}{x}}_{\text{start}-\text{end}}$ = 5.79–2.55, *SE* = 1.01–0.69), followed by pale crabs on “mud” background (${\overset{¯}{x}}_{\text{start}-\text{end}}$ = 13.01–5.04, *SE* = 2.39–0.83), dark crabs on “rock” background (${\overset{¯}{x}}_{\text{start}-\text{end}}$ = 13.31–15.39, *SE* = 1.82–0.66) and pale crabs on “rock” background (${\overset{¯}{x}}_{\text{start}-\text{end}}$ = 10.91–20.93, *SE* = 1.97–1.80). The closest chromatic match was achieved by dark crabs on “mud” background (${\overset{¯}{x}}_{\text{start}-\text{end}}$ = 3.60–1.07, *SE* = 0.27–0.33), but followed by dark crabs on “rock” background (${\overset{¯}{x}}_{\text{start}-\text{end}}$ = 1.98–2.88, *SE* = 0.41–0.39), pale crabs on “mud” background (${\overset{¯}{x}}_{\text{start}-\text{end}}$ = 2.67–2.94, *SE* = 0.26–0.24) and pale crabs on “rock” background (${\overset{¯}{x}}_{\text{start}-\text{end}}$ = 2.06–3.09, *SE* =0.20–0.76). Thus, there was limited evidence of background‐specific matching and this only occurred on mudflat background, as crabs did not improve match to the rock background under the fish vision model.

**Table 2:** Linear mixed‐effects analyses (LMER) testing the background matching of crabs

Subject	Estimate	*SE*	*df*	*t*‐Value	*p*
Luminance match (JND)					
(Intercept)^a^	9.59	1.29	108.4	7.41	<0.001
Background [rock pool]	2.65	1.89	110.1	1.39	0.164
Shade [pale]	5.32	2.02	112.7	2.63	0.009
Time [week]	−0.46	0.14	523.2	−3.22	0.001
Background × Shade	−10.14	2.84	110.2	−3.56	<0.001
Background × Week	0.16	0.21	525.2	0.79	0.426
Shade × Week	−0.73	0.24	533.1	−2.99	0.002
Background × Shade × Week	1.33	0.32	527.7	4.05	<0.001
Chromatic match (JND)					
(Intercept)^a^	3.58	0.27	11.4	13.25	<0.001
Background [rock pool]	−1.98	0.35	77.2	−5.63	<0.001
Shade [pale]	−1.07	0.37	78.2	−2.87	0.005
Time [week]	−0.19	0.01	518.6	−9.85	<0.001
Background × Shade	1.21	0.52	77.4	2.29	0.024
Background × Week	0.32	0.02	519.6	11.12	<0.001
Shade × Week	0.14	0.03	523.7	4.23	<0.001
Background × Shade × Week	−0.14	0.04	520.8	−3.16	0.001

Note

The match is determined using a fish vision model. LMER predicts the luminance and chromatic match measured as JNDs (i.e., just noticeable differences) response in relation to crab shading (“shade”), rearing background type (“background”), time (“week”) and their interactions. Intercept includes rearing tank and crab ID as random variables.

a Intercept includes factor level: Background [mud] and Shade [dark].

### 3.2 Consequences of phenotype on detection and survival

**Figure 3:**
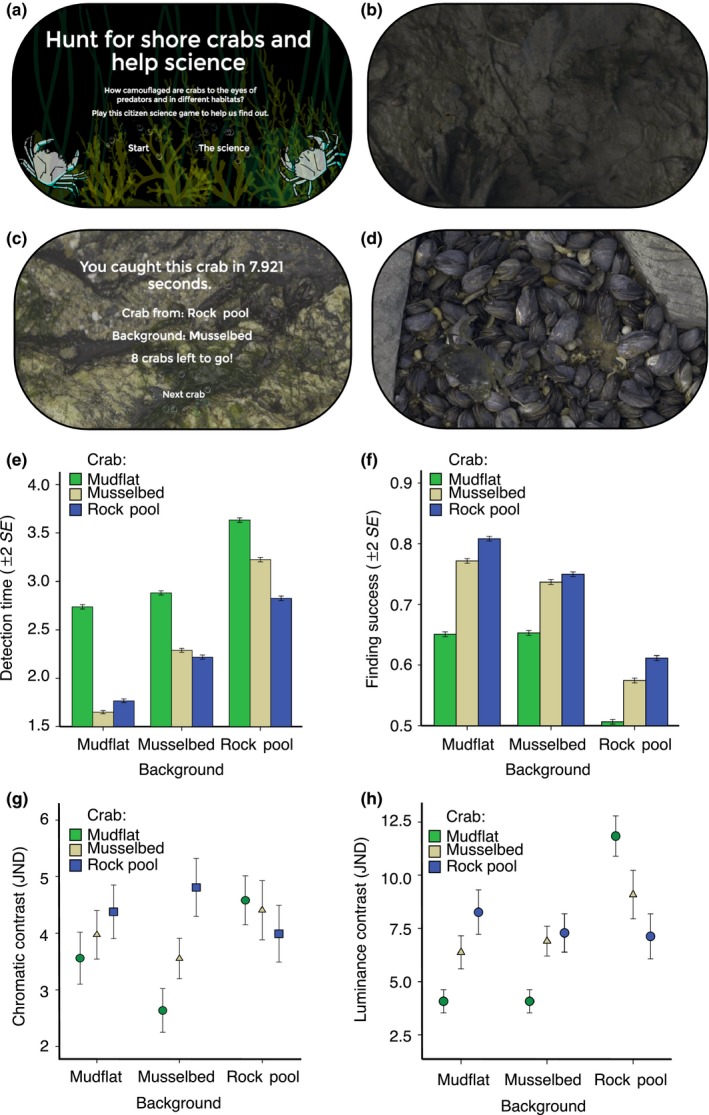
Computer‐based detection experiment. We used a citizen science game (a), based at the Natural History Museum in London, UK, where subjects searched for hidden crabs on a touch screen and detection times were measured. People were instructed to find crabs as quickly as possible from varied background types: mudflats (b), rock pools (c) and mussel beds (d). In citizen science experiment, crabs picked from mudflats, mussel beds and rock pools were presented against their own and other habitat types on touch screen. The bar plots illustrate which crabs are hardest to find (detection time, [e], in seconds to spot the crab from a background) and thus have the highest survival benefit hiding in three major tidal habitats (finding success, [f], as the proportion of successful clicks of particular crab type presented against different backgrounds). Receptor noise‐limited human vision model predicts that chormatic contrasts of all crabs were reasonably hard (i.e., <5 JNDs) to detect in the game (g), whereas luminance differences were larger and rendered some, except “mudflat crabs”, easier to find (h). Error bars show ± 2 SE

Next, we undertook a large‐scale computer “citizen science” experiment (Figure 3), where human subjects (“predators”) searched for hidden crabs from different origins against variable background types on a touch screen. The data consist of 472,961 individual clicks from 19,102 games played. In accordance with our expectations, crabs were harder to find against visually more complex backgrounds (Figure 3, Table 3). The average time to find the crabs was 3.24 s (*N* = 144,974, *SD* = 2.82) on rock pools, 2.47 s (*N* = 148,937, *SD* = 2.38) on mussel beds and 2.08 s (*N* = 179,096, *SD* = 2.24) on mudflat backgrounds. This mirrors decreasing visual complexity of the background, and thus, decrease in signal‐to‐noise ratio in prey detection.

**Table 3:** Linear mixed‐effects analyses (LMER) testing the efficacy of camouflage

Subject	Estimate	*SE*	*df*	*t*‐Value	*p*
(Intercept)^a^	2,338.03	73.23	436	31.92	<0.001
Crab Habitat [mussel]	−893.08	142.06	310	−6.28	<0.001
Crab Habitat [pool]	−1,078.90	65.65	4,292	−16.43	<0.001
Photo Habitat [mussel]	239.71	12.28	509,442	19.51	<0.001
Photo Habitat [pool]	727.28	11.87	510,886	61.28	<0.001
Crab [mussel] × Photo [mussel]	225.26	17.82	508,001	12.63	<0.001
Crab [pool] × Photo [mussel]	161.08	17.91	508,139	8.99	<0.001
Crab [mussel] × Photo [pool]	453.92	17.22	509,449	26.36	<0.001
Crab [pool] × Photo [pool]	109.53	17.39	509,349	6.29	<0.001

Note

Here, under the test was how quick crabs were to find (i.e., camouflage efficacy) against background types. LMER predicts the time to find crab (i.e., latency to click) risk in relation to crab origin (“crab habitat”), background habitat displayed (“photo habitat”) and their interaction. Intercept includes game ID and crab size as random variables.

a Intercept includes factor level: Crab [mud] and Photo [mud].

Surprisingly, crabs were not hardest to find against their original habitat type as we predicted, but instead, the mudflat crab type (i.e., dark green phenotype) was hardest to spot against all backgrounds (Figure 3, Table 4). The average time to find mudflat type crabs was 3.11 s (*N* = 171,103, *SD* = 2.75), followed by mussel bed type crabs with 2.45 s (*N* = 153,937, *SD* = 2.44) and rock pool type crabs with 2.31 s (*N* = 147,967, *SD* = 2.39). Overall, there was no significant difference in how quickly predators could find prey in trichromatic (*N* = 240,265, mean = 2.57, *SD* = 2.53) or dichromatic (*N* = 232,742, mean = 2.72, *SE* = 2.61) simulated “worlds,” so visual system was omitted from the final models.

**Table 4:** Generalized linear mixed‐effects analyses (GLMM) testing the efficacy of camouflage

Subject	Estimate	*SE*	*Z*‐value	*p*
(Intercept)^a^	2.32	0.09	25.13	<0.001
Crab Habitat [mussel]	1.08	0.16	6.55	<0.001
Crab Habitat [pool]	1.47	0.09	15.65	<0.001
Photo Habitat [mussel]	−0.18	0.01	−9.43	<0.001
Photo Habitat [pool]	−0.93	0.01	−51.03	<0.001
Crab [mussel] × Photo [mussel]	−0.42	0.03	−14.22	<0.001
Crab [pool] × Photo [mussel]	−0.39	0.03	−12.74	<0.001
Crab [mussel] × Photo [pool]	−0.61	0.02	−21.83	<0.001
Crab [pool] × Photo [pool]	−0.37	0.02	−12.88	<0.001

Note

Here, under the test was the success (i.e., crab survival) to locate crabs correctly against background types. GLMM predicts the success to locate crabs correctly in relation to crab origin (“crab habitat”), background habitat displayed (“photo habitat”) and their interaction. Intercept includes game ID and crab size as random variables.

a Intercept includes factor level: Crab [mud] & Photo [mud].

To investigate chromatic and luminance discrimination values (i.e., crab detectability to humans), we ran another set of analyses using LMER. In both luminance (*F*_4,905_ = 40.22, *p* < 0.001) and chromatic matching (*F*_4,904_ = 36.86, *p* < 0.001), there was a significant two‐way interaction between background against which the crab was presented and crab origin (Table 5, Figure 3 *). *Discrimination values were significantly different between background types, but this was varied with respect to crab origin (especially against mussel beds). Chromatic camouflage of crabs was generally good (<5 JNDs) across all comparisons, but mudflat crabs were better matched to the luminance (i.e., lightness) of the backgrounds apart from rock pool background where they appeared darker than the generic rock pool background (Figure 3).

**Table 5:** Linear mixed‐effects analyses (LMER) testing the background matching of crabs in the citizen science game

Subject	Estimate	*SE*	*df*	*t*‐Value	*p*
Luminance match (JND)					
(Intercept)^a^	8.91	2.11	37	4.21	<0.001
Background [musselbed]	4.73	0.95	904	4.94	<0.001
Background [rock pool]	16.16	1.01	904	15.94	<0.001
Crab [musselbed]	4.75	2.92	37	1.62	0.112
Crab [rock pool]	8.37	2.92	37	2.86	<0.001
Background [mb] × Crab [mb]	−3.70	1.32	904	−2.79	<0.001
Background [rp] × Crab [mb]	−10.62	1.40	904	−7.57	<0.001
Background [mb] × Crab [rp]	−7.35	1.33	905	−5.50	<0.001
Background [rp] × Crab [rp]	−17.80	1.41	905	−12.57	<0.001
Chromatic match (JND)					
(Intercept)^a^	1.83	0.21	35	8.41	<0.001
Background [musselbed]	−0.89	0.08	904	−10.32	<0.001
Background [rock pool]	0.21	0.09	904	2.32	0.019
Crab [musselbed]	0.10	0.30	36	0.36	0.721
Crab [rock pool]	−0.07	0.30	36	−0.23	0.813
Background [mb] × Crab [mb]	0.34	0.12	904	2.89	0.003
Background [rp] × Crab [mb]	−0.16	0.12	904	−1.26	0.207
Background [mb] × Crab [rp]	1.00	0.12	904	8.31	<0.001
Background [rp] × Crab [rp]	−0.41	0.13	904	−3.27	<0.001

Note

LMER predicts the luminance and chromatic match measured as JNDs (i.e., just noticeable differences) response in relation to crab origin (“crab”) and background type where presented (“background”). Intercept includes crab ID as random variable.

a Intercept includes factor level: Background [mud] and Crab origin [mud].

## 4 DISCUSSION

**Figure 4:**
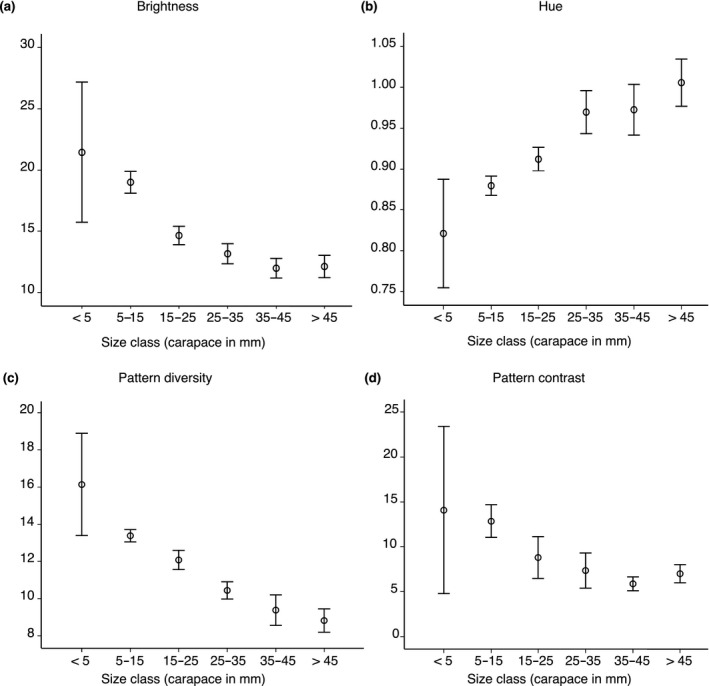
Ontogenetic colour change in the green shore crab (*Carcinus maenas*) in the field. The data are derived from large‐scale field monitoring study by Nokelainen, Hubbard et al. (2017). The figure shows the change in carapace colour over time obtained from avian vision model cone catch data. The panels show decreases in brightness (a), bias towards medium wavelengths as hue (b) as well as loss of pattern diversity (c) and contrast (d) as crabs grow. The combined effects of red and increases in green channel apparently drive the ontogenetic colour change. Y‐axes show 95% CI

We show that ontogenetic changes in coloration can facilitate improvement in camouflage and thus alter predation risk in shore crabs. Importantly, our results are in direct accordance with findings in the field (Figure 4, Supporting Information Figure S3), where crabs are also more green, increasingly uniform and darker with age (Nokelainen, Hubbard et al., 2017; Stevens et al., 2014b). Thus, our study shows how mechanisms of colour change and adaptive value of camouflage underly how the phenotypes of wild animals change with age/size. Changes in crab appearance with age do not come via specialization to particular habitat types (as would be expected if plasticity is key), but rather, through a more generalist background resemblance (consistent with ontogenetic change). This shows the ability of wild animals to tune their camouflage through development in a manner that promotes survival.

In the laboratory experiment, juvenile crabs developed a dull green/brown coloration with reduced patterning over time regardless of background type, which indicates a long‐term (i.e., occurring over weeks) change in coloration through ontogeny (Bedini, 2002; Reid et al., 1997; Styrishave et al., 2004; Todd et al., 2009). We predicted that crabs would develop a coloration that would improve their background match through colour change and plasticity (Iampietro, 1999; Stevens et al., 2014b, 2013). Specifically, juvenile crabs have been shown to be able to change their brightness in accordance with the background over hours and days (Powell, 1964; Stevens et al., 2014a), and weeks (Stevens, 2016). In contrast, we found that only crabs reared on the “mudflat” background improved their match over several weeks. Earlier work has repeatedly reported that wild adults are more uniform, green and darker in appearance than juveniles (Crothers, 1968; Hogarth, 1978; McGaw, Kaiser, Naylor, & Hughes, 1992; Nokelainen, Hubbard et al., 2017; Reid et al., 1997; Stevens et al., 2014b; Styrishave et al., 2004; Todd et al., 2006). Low chromatic variability in adult crabs could also be partly a result of physiological constraints as larger crabs must invest more on reproductive structures and carapace strength rather than to maintenance of chromatic variability in protective coloration (Anderson et al., 2013). In accordance, the analysis of carapace brightness revealed that crabs became darker over time and developed coloration towards the medium (green) wavelengths. Our results also showed that the crabs developed more uniform patterning (see also Figure S2). It is not well known what maintains the high colour variation in juvenile crabs, but it may be related to the need to match variable background habitats at spatial scales (Nokelainen, Hubbard et al., 2017) that are relevant when individuals are small, and/or breaking predator search image formation (Bond & Kamil, 2002; Duarte et al., 2017; Karpestam et al., 2014; Punzalan et al., 2005). It is plausible that juvenile crabs may also rely on other types of camouflage, such as disruptive coloration (Todd et al., 2006), and this may be habitat‐specific, with crabs from rock pools favouring disruption and crabs from mudflats tending towards background matching.

In the detection experiments, we expected that visual complexity of the background would increase the detection times to find the prey (Merilaita, 2010; Rosenholtz, Li, & Nakano, 2007; Troscianko, Lown, Hughes, & Stevens, 2013). This is because increasing background complexity decreases the signal‐to‐noise ratio that predators must process in order to detect prey (Endler, 1992; Merilaita, Scott‐Samuel, & Cuthill, 2017). Correspondingly, crabs were easiest to find from more homogeneous mudflat background followed by polychromatic mussel beds and hardest to find in more heterogeneous rock pools. This suggests that selection for camouflage may be more intense in simple visual scenes. We also predicted that crabs would be hardest to find when placed against their original habitat type, because this would support a substrate‐specific (or specialist) background matching hypothesis (Carvalho‐Batista et al., 2015; Detto, Hemmi, & Backwell, 2008; Krause‐Nehring et al., 2010; Stevens et al., 2013). In contrast, the mudflat crabs characterized by the dark green phenotype were hardest to find against all background types. Thus, it appears that dark green shore crabs are well suited for maintaining camouflage on a variety background. Some caution is needed in interpreting the results of the computer experiments since humans are not the natural predators of these crabs. However, conducting predation experiments with this highly mobile species in the intertidal environment is challenging, and natural predators are varied, including various fish and bird species, among other taxa (Crothers, 1968), that vary in visual ability from mono‐, to di‐, tri‐ and tetrachromatic colour vision and a range of spatial acuities. Here, humans offer a reasonable middle ground (being trichromats) and are strongly visually guided. As such, our results using humans as visually guided predators should be broadly representative to provide information about relative importance of colour patterns that influence detection in the wild (Karpestam, Merilaita, & Forsman, 2013), but work with natural predators is needed.

In combination, our detection experiment showed that more uniform green coloration provided effective camouflage in all habitats, and our experiment showed that this phenotype arises in at least the substrates tested here. This fits with the common observation that many subadult and adult shore crabs are uniform green/brown in the wild (Amaral, Cabral, Jenkins, Hawkins, & Paula, 2009; Crothers, 1968; Nokelainen, Hubbard et al., 2017; Reid et al., 1997; Stevens et al., 2014a; Todd et al., 2006). There are several explanations for why a progression to a more uniform green appearance with age may be selected. First, the three habitats we tested in the computer experiments may all have had sufficient numbers of patches resembling green crabs to facilitate camouflage, whereas more complex patterns may have only resembled a small number of the highly variable patches in the rock pool and mussel bed habitats. Thus, older individuals may have a higher chance of survival across a range of background types with a generalist appearance arising through ontogeny providing some camouflage in each habitat, even if not optimally tuned to all of them (Dimitrova & Merilaita, 2014; Houston, Stevens, & Cuthill, 2007; Merilaita, Lyytinen, & Mappes, 2001). In addition, adult crabs are known to be mobile (Edwards, 1958; Roman & Palumbi, 2004), meaning that they require a more generalist camouflage with increasing age/size, and there is also evidence that as shore crabs age that they move into deeper waters (McGaw et al., 1992), where it is possible that these habitats have a greater abundance of dull backgrounds. In contrast, juvenile crabs are often more abundant in nursery sites (Amaral et al., 2009; Stevens et al., 2014b) and often face visual backgrounds of different spatial scales relative to body size. Juvenile crabs from rock pools, for example, tend to be diverse in appearance (Nokelainen, Hubbard et al., 2017; Stevens et al., 2014b) and may rely on other types of camouflage such as disruptive coloration and resembling small markings. In rock pool sites, owing to their high variability in background patches, matching many of these specific patches may be an ineffective strategy overall. Size‐related habitat and colour shifts may have important fitness consequences for crabs, as growth and survival are both improved in the new habitat (Hultgren & Stachowicz, 2008, 2010, 2011). This may be less effective when of a larger size and more mobile over a range of backgrounds. Finally, in nursery habitats, such as rock pools, the variability of crabs may be beneficial as it may impair predator search image formation (Bond, 2007). Overall, ontogenetic changes in shore crabs may facilitate age‐ and habitat‐dependent camouflage (Todd et al., 2009), as well as offering a good general solution to environmental diversity.

Taken together, our results help explain why so many animals (e.g., snakes, lizards, crabs) all develop a similar coloration over ontogeny. Phenotypic surveys in the field at multiple spatial scales across habitats show strong associations between aspects of appearance and substrate type (Boratynski, Brito, Campos, Karala, & Mappes, 2014; Nokelainen, Hubbard et al., 2017; Stevens et al., 2015; Todd et al., 2012). While work has yet to quantify how this translates into actual camouflage match, the implication is that many animals show substrate‐specific camouflage across habitats and local patches. This is seemingly in contrast with the results here. However, there is growing evidence in many animal taxa including crabs that individuals of different appearance from within a species choose where to rest in order to improve camouflage in their respective habitats (Kang, Moon, Lee, & Jablonski, 2012; Kettlewell & Conn, 1977; Kjernsmo & Merilaita, 2012; Lovell, Ruxton, Langridge, & Spencer, 2013; Marshall, Philpot, & Stevens, 2016; Sargent, 1966; Uy et al., 2017; reviewed by Stevens & Ruxton, 2018). Otherwise, it is hard to explain very local level phenotype‐substrate associations of crabs without the role of behavioural background selection (Nokelainen, Hubbard et al., 2017; Nokelainen, Stevens, & Caro, 2017; Todd et al., 2012). Concurrently, ontogenetic changes may facilitate a generalist camouflage and appear to be linked to changes that would, on average, give the biggest survival advantage. The appearance of animals in the wild, and changes associated with age and habitat, likely reflects a complex interplay between genetics, plasticity and ontogeny, underpinned by a variety of mechanisms and maintained by multiple selective pressures. Overall, the evolution of camouflage can be better understood by wider considerations of how the optimal phenotype to hide from predators may change over the life history of animals.

## AUTHORS' CONTRIBUTIONS

O.N. wrote the first draft of the manuscript, designed experiments and analysed data; R.M. collected common garden data; S.M. and N.P. contributed on citizen science game; and M.S. contributed substantially to the project design and manuscript editing.

## Data Availability

The data are archived (http://urn.fi/URN:NBN:fi:jyu-201901071081) at the repository of University of Jyväskylä (https://jyx.jyu.fi).
