# Supplementary material for: Improved camouflage through ontogenetic colour change confers reduced detection risk in shore crabs
Source: Funct Ecol. 2019 Jan 24;33(4):654–69. doi: 10.1111/1365-2435.13280 (PMC6559319; doi:10.1111/1365-2435.13280)
Supplement: Supplementary file 1 [file FEC-33-654-s001.pdf]

Functional Ecology

BRITISH ECOLOGICAL SOCIETY

Shore crabs change colour with age to blend into their surroundings

Ossi Nokelainen, Ruth Maynes, Sara Mynott, Natasha Price & Martin Stevens

Many animals change appearance with age but the reasons why are rarely tested. Common shore crabs (*Carcinus maenas*), for example, are known for their ability to change colour over time. Young crabs show remarkable variation in coloration and it has been suggested that their variable appearance may help them to hide from predators in the habitats they use. However, as crabs grow they become more mobile and adult crabs, in contrast, are known to possess a more uniform coloration. This creates a problem: how to remain hidden in habitats that are variable and very different in appearance?

To answer this, we first reared young shore crabs of two shades, pale or dark, on two background types resembling different habitats for 10 weeks. We predicted that crabs would adopt a coloration that would improve their match to the background, but instead they all developed a dark green or brown appearance. Next, we undertook an experiment at the Natural History Museum London during the Colour and Vision exhibition, where visitors searched for crabs representing natural colour variation from different habitats. Remarkably, crabs were not hardest to find against their original habitat, but instead the dark green or 'mudflat crabs' were hardest to detect against all backgrounds.

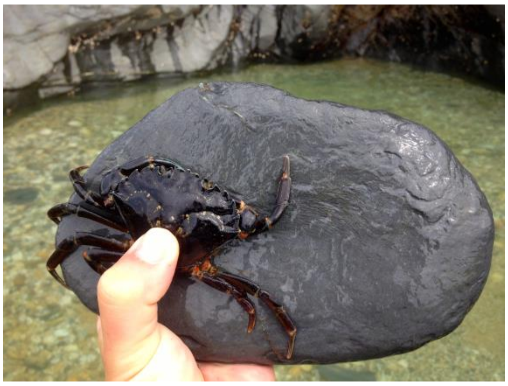

Adult Carcinus maenas

These findings suggest that shore crabs change into similar colour with age, which appears to be a good general solution to match several habitat types and therefore to hide from predators. We conclude that the evolution of camouflage can be better understood by recognizing that the optimal appearance to hide may change over the lifespan of many animals.
