# Supplementary material for: Improved camouflage through ontogenetic colour change confers reduced detection risk in shore crabs
Source: Funct Ecol. 2019 Jan 24;33(4):654–69. doi: 10.1111/1365-2435.13280 (PMC6559319; doi:10.1111/1365-2435.13280)
Supplement: Supplementary file 2 [file FEC-33-654-s002.pdf]

# Supplementary material for Nokelainen et al. 2019 Functional Ecology

**Title:** Improved camouflage through ontogenetic colour change confers reduced detection risk in shore crabs

**Authors:**

Ossi Nokelainen[1,2*], Ruth Maynes[1], Sara Mynott[1], Natasha Price[1] & Martin Stevens[1*]

[1]Centre for Ecology and Conservation, College of Life and Environmental Science, University of Exeter, TR10 9FE Penryn, UK

[2]Department of Biological and Environmental Science, University of Jyväskylä, P.O. Box 35, 40014 University of Jyväskylä, Finland

[*] Authors for correspondence (ossi.nokelainen@jyu.fi, martin.stevens@exeter.ac.uk)

**Contents:** Page

Supplementary tables

Table S1 2

Table S2 6

Table S3 7

Supplementary figures

Figure S1 9

Figure S2 10

Figure S3 11

Supplementary references 12

Table S1: Details on photography, vision modelling, pattern analysis and background match estimation.

---

*Photography and vision modelling*

Photography, initial image calibration and analysis broadly followed previously used methods (Stevens et al. 2014a). Imaging was undertaken with Samsung NX1000 digital camera converted to full spectrum with no quartz filter to enable UV sensitivity, and fitted with a Nikon EL 80 mm lens. For the human visible photos, we placed a UV and infrared (IR) blocking filter in front of the lens, which transmits wavelengths only between 400 – 680 nm (Baader UV/IR Cut Filter). For the UV images, a UV pass and IR blocking filter was used (Baader U filter), which transmits between 320-380 nm. We have previously characterized the spectral sensitivity of our cameras (Troscianko and Stevens 2015). For calibration purposes, each photograph included a grey reflectance standard, which reflects light equally at 7% and 93% between 300 and 750 nm.

Images were taken in RAW format with manual white balance and a fixed aperture setting. During calibration (in Image J; Troscianko & Stevens 2015), images were converted to uncompressed TIFF files and the images of each crab comprised four bandpass layers corresponding to the long-wavelength (LW), medium-wavelength (MW), short-wavelength (SW), and UV parts of the spectrum. To control for nonlinear responses in image value to changes in light levels, we linearized all images based on quantified camera responses to a set of eight Spectralon grey standards with reflectance values ranging from 2–99% (Stevens et al. 2007, 2009). We controlled for differences in ambient light by standardizing (equalizing) the images to the grey standard, and scaled each image channel to reflectance, where an image value of 255 on an 8-bit scale equals 100%

reflectance (Stevens et al. 2007, Troscianko and Stevens 2015). These images therefore corresponded to the reflectance properties of the crab patterns in four parts of the spectrum and could be used for analysis of coloration and pattern. For each image we measured the entire dorsal side of the crab carapace.

We modelled predator vision according to fish visual sensitivity, because fish are major predators of juvenile shore crabs (Crothers 1968). We used the spectral sensitivities of pollack (*Pollachius pollachius*) adults (Shand et al. 1988) to generate cone catch values for fish predators based on pigment model (Govardovskii et al. 2000) incorporating the lens transmission data (Troscianko and Stevens 2015). The cone mapping procedure converts images from camera reflectance space to animal receptor values and is highly accurate compared to alternative methods using reflectance spectrometry (Cuthill et al. 2006, Pike 2011, Stevens et al. 2014a, Troscianko and Stevens 2015).

---

*Pattern analysis procedure*

The pattern analysis technique (a 'granularity' analysis) involves decomposing an image into a series of different spatial frequencies ('granularity bands') using Fourier analysis and band pass filtering, followed by determining the relative contribution of different marking sizes to the overall pattern (Barbosa et al. 2008, Hanlon et al. 2009, Stoddard and Stevens 2010). The filtering into different frequency bands functions like a sieve, capturing information at different spatial scales corresponding to different sized markings. Pattern analysis was conducted in custom files for Image J (Troscianko and Stevens 2015), with analysis based on different pixel sizes. The analysis calculates the amount of information, or energy, corresponding to markings of different sizes, starting

with small markings (few pixels) and increasing in size to larger markings. For each granularity band, we calculated the pattern 'energy', being the sum of the squared pixel values in each image divided by the total number of pixels (Barbosa et al. 2008, Chiao et al. 2009, Stoddard and Stevens 2010). The energy values across all filtered images produce a 'granularity spectrum', being a plot of energy versus pixels (marking size). From each granularity spectrum we obtained crab carapace pattern information as described below (Stoddard and Stevens 2010).

---

*Crab phenotype determination and background match*

To characterize crab phenotype, we analysed the data both with normalised camera responses and fish vision modelled data. We calculated five measures of crab appearance based on colour and pattern metrics using previously published methods (Barbosa et al. 2008, Hanlon et al. 2009, Stoddard and Stevens 2010, Stevens et al. 2014b). 1) Luminance: the perceived lightness based on the LW receptor in fish and LW channel in normalised camera responses. 2) Hue: calculated as a ratio of shortwave versus longwave receptor responses (e.g. (SW/ LW) (Komdeur et al. 2005, Spottiswoode and Stevens 2011). 3) Proportion energy (i.e. how much one marking size dominates, or the diversity of marking sizes), being the proportion of the total energy across the entire spectrum corresponding to the maximum energy point, with a high value indicating that the pattern is dominated by one or a few marking sizes. 4) Total energy (i.e. pattern contrast), being the total energy of the spectrum (Chiao et al. 2009), whereby higher values indicate more contrasting markings. 5) Marking size (i.e. carapace marking size), with the maximum energy value at any point in the spectrum corresponding to the dominant marking size.

To examine the level of background match, we calculated how changes in the crab carapace influenced their level of match to the experimental backgrounds. To do so we used a visual discrimination model (Vorobyev et al. 1998), which is based on differences in colour or luminance based on photo catch values. We used a Weber fraction value of 0.05 for the most abundant cone type (Govardovskii et al. 2000) with receptor cone ratios SW 168 and LW 339 for the pollack vision. The model yields values in 'just noticeable differences' (JNDs), whereby differences between 1 and 3 are interpreted that two stimuli are unlikely to be discriminated by an observer (and hence indicate a good background match), and larger values are increasingly likely to be discriminable (Siddiqi et al. 2004).

Table S2: Linear mixed effects analyses (LMER) testing the luminance and colour change of green shore crabs over time. LMER predicts the luminance and hue changes as a response to crab original appearance (shade), rearing background type (background), time (week) and their interactions. Intercept has crab ID and tank as random variables.

| Subject | Estimate | s.e. | DF | t-value | P |
|---|---|---|---|---|---|
| Luminance | | | | | |
| (Intercept)° | 13.80 | 1.41 | 21.7 | 9.77 | <0.001 |
| Background [rock pool] | 0.44 | 1.73 | 53.9 | 0.25 | 0.798 |
| Shade [pale] | 7.29 | 2.08 | 89.0 | 3.50 | <0.001 |
| Time [week] | -0.32 | 0.13 | 531.5 | -2.36 | 0.018 |
| Background * Shade | 3.68 | 2.61 | 54.8 | 1.41 | 0.163 |
| Shade * Week | 0.14 | 0.20 | 530.9 | -5.31 | <0.001 |
| Hue | | | | | |
| (Intercept)° | 1.17 | 0.02 | 76.5 | 49.01 | <0.001 |
| Background [rock pool] | -0.03 | 0.03 | 74.3 | -1.00 | 0.319 |
| Shade [pale] | 0.03 | 0.03 | 58.0 | 1.08 | 0.281 |
| Time [week] | 0.01 | 0.01 | 527.8 | 9.12 | <0.001 |
| Background * Shade | -0.01 | 0.04 | 57.0 | -0.18 | 0.853 |
| Background * Week | -0.01 | 0.01 | 525.2 | -1.00 | 0.314 |

Intercept includes factor level: Background [mud] & Shade [dark].

Table S3: Linear mixed effects analyses (LMER) testing the changes in carapace patterning over time. LMER predicts the carapace pattern change in relation to crab original appearance (shade), rearing background type (background), time (week) and their interactions. Intercept has crab ID and rearing tank as random variables.

| Subject | Estimate | s.e. | DF | t-value | P |
|---|---|---|---|---|---|
| Pattern diversity | | | | | |
| (Intercept)° | 1.450e-01 | 2.984e-02 | 2.100e+00 | 4.860 | 0.036 |
| Background [rock pool] | 1.320e-02 | 7.528e-03 | 1.124e+02 | 1.754 | 0.082 |
| Shade [pale] | 2.589e-02 | 8.029e-03 | 1.151e+02 | 3.225 | 0.001 |
| Time [week] | 4.049e-04 | 5.790e-04 | 5.264e+02 | 0.699 | 0.484 |
| Background * Shade | -1.252e-02 | 1.131e-02 | 1.124e+02 | -1.107 | 0.270 |
| Background * Week | -1.517e-03 | 8.558e-04 | 5.281e+02 | -1.772 | 0.076 |
| Shade * Week | -4.201e-03 | 9.806e-04 | 5.362e+02 | -4.285 | <0.001 |
| Background * Shade * Week | 3.531e-03 | 1.319e-03 | 5.308e+02 | 2.677 | 0.007 |
| Pattern contrast | | | | | |
| (Intercept)° | 3537.47 | 787.92 | 2.70 | 4.49 | <0.001 |
| Background [rock pool] | 131.61 | 475.23 | 54.50 | 0.27 | 0.780 |
| Shade [pale] | 1170.15 | 530.67 | 67.20 | 2.20 | 0.030 |
| Time [week] | -112.19 | 23.28 | 550.50 | -4.81 | <0.001 |
| Background * Shade | 1201.02 | 715.42 | 54.80 | 1.67 | 0.090 |
| Background * Week | -285.63 | 35.31 | 550.40 | -8.08 | <0.001 |

Table S3 continued

| | | | | | |
|---|---|---|---|---|---|
| Marking size | | | | | |
| (Intercept)° | 150.13 | 27.641 | 19.30 | 5.431 | <0.001 |
| Background [rock pool] | -44.01 | 36.31 | 213.4 | -1.21 | 0.226 |
| Shade [pale] | 21.21 | 38.89 | 219.3 | 0.54 | 0.586 |
| Time [week] | 10.31 | 3.63 | 534.4 | 2.836 | 0.004 |
| Background * Shade | -31.28 | 54.57 | 213.0 | -0.57 | 0.567 |
| Background * Week | -1.71 | 5.37 | 537.2 | -0.31 | 0.750 |
| Shade * Week | -15.37 | 6.11 | 550.5 | -2.51 | 0.012 |
| Background * Shade * Week | 17.60 | 8.26 | 541.9 | 2.131 | 0.033 |

Intercept includes factor level: Background [mud] & Shade [dark].

Figure S1: Comparison between artificial and natural backgrounds. Here the match of our artificial backgrounds to natural ones was compared using a photographic data. Briefly, we quantified similarity of the backgrounds in trichromatic RGB colour space, based on reflectance data, to quantify brightness (A) and hue (B).

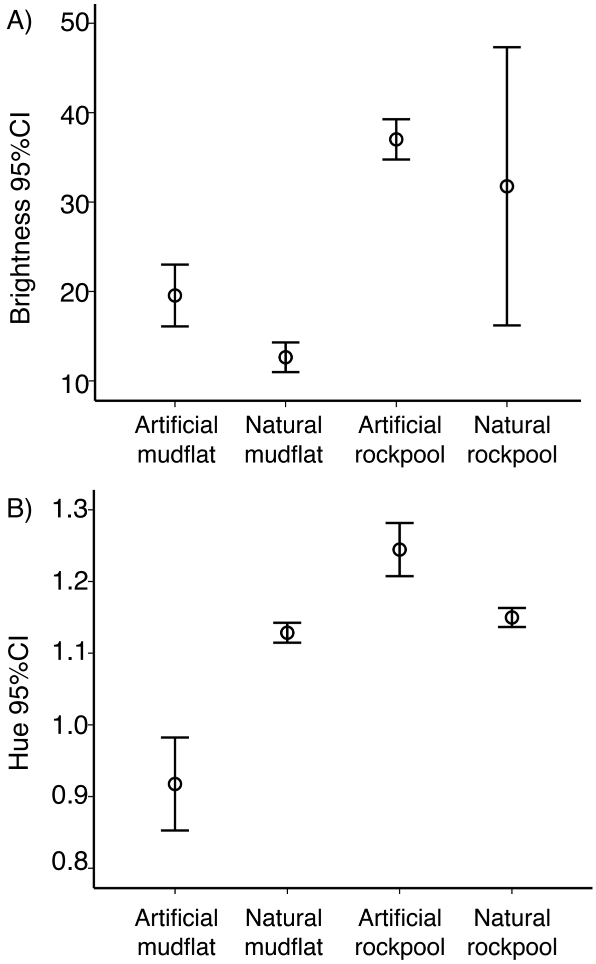

Figure S2: Ontogenetic colour change in the green shore crab (*Carcinus maenas*). The figure shows the change in carapace colour over time obtained from normalised camera responses. The panels show different wavelenght channels: red (A), green (B), blue (C) and UV (D). The time is shown on x-axis and the mean colour responses (i.e. the relative expression of the channel as 0-100) on y-axis. The lines are different treatment groups as follows. Solid green: dark-shaded crabs on mud background (DAMD); Solid blue: dark-shaded crabs on rock pool background (DARO); Dashed green: pale-shaded crabs on mud background (PAMD); Dashed blue: pale-shaded crabs on rock pool background (PARO). Notice that the expression of green wavelengths shows mean increase over time in relation to other wavelengths. The combined influence of predominant red and increases in green channel (as well as decreases in blue and low expression of UV) apparently drives the ontogenetic colour change causing the 'mudflat' phenotype.

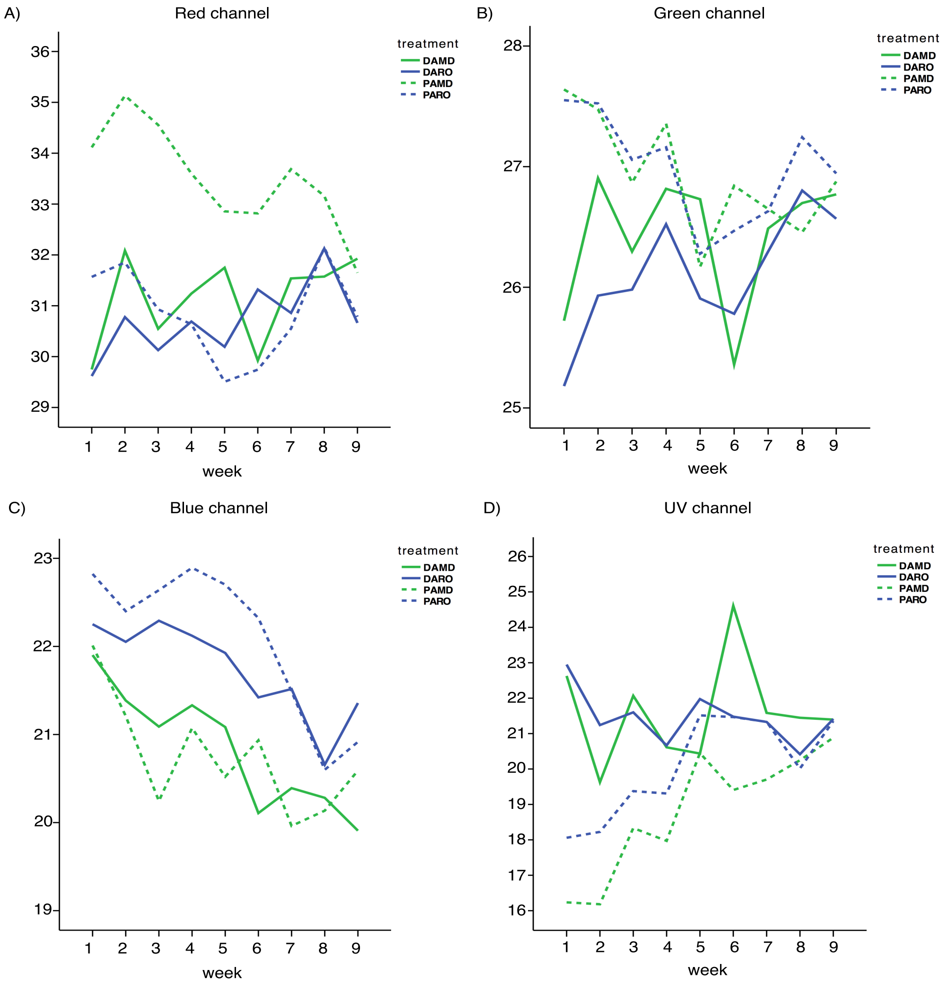

Figure S3: Ontogenetic colour change in the green shore crab (*Carcinus maenas*) in the field. The data is derived from large-scale field monitoring study (Nokelainen et al. 2017). The figure shows the change in carapace colour over time obtained from avian vision model cone catch data in relation to the catching habitat of the crab. The panels show decreases in long wavelengths (A), whereas there is relative increase of medium (B) or short (C) wavelengths and again decrease in UV wavelengths (D) as crabs grow. The same pattern is seen through all habitat types here.

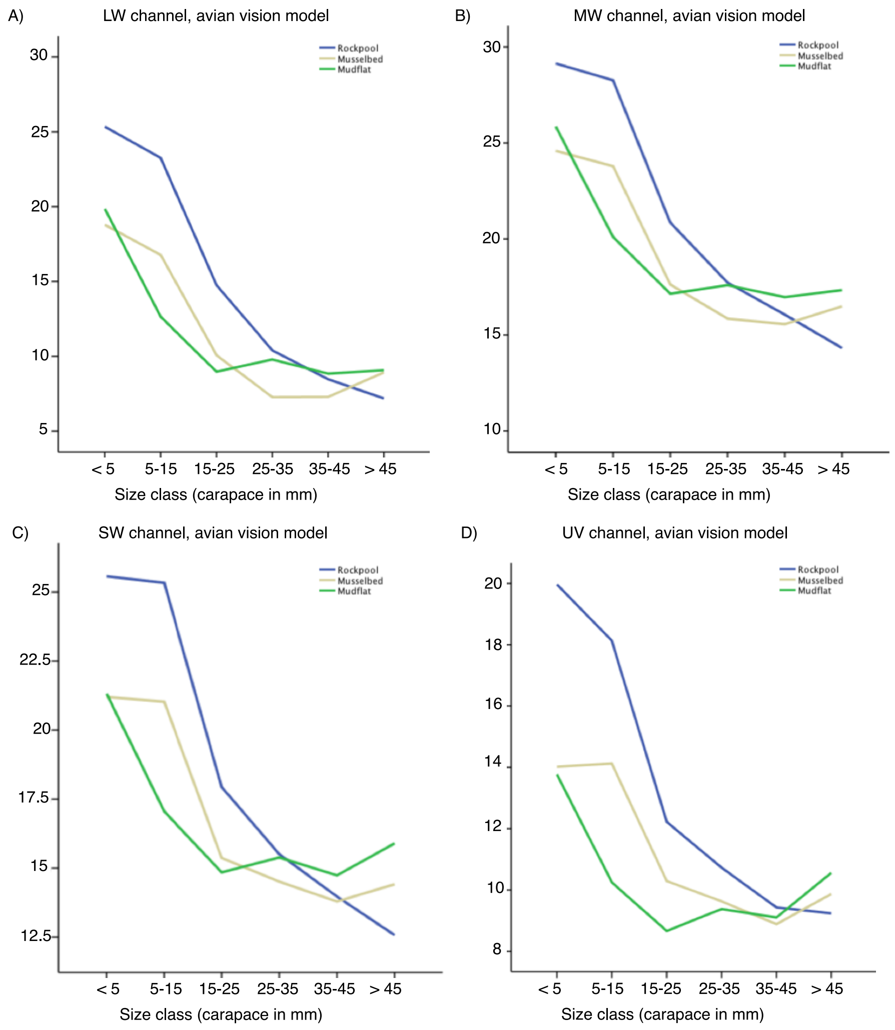

**References**:

Barbosa, A., L. M. Mäthger, K. C. Buresch, J. Kelly, C. Chubb, C. C. Chiao, and R. T. Hanlon. 2008. Cuttlefish camouflage: The effects of substrate contrast and size in evoking uniform, mottle or disruptive body patterns. Vision Research 48:1242–1253.

Chiao, C. C., C. Chubb, K. Buresch, L. Siemann, and R. T. Hanlon. 2009. The scaling effects of substrate texture on camouflage patterning in cuttlefish. Vision Research 49:1647–1656.

Crothers, J. H. 1968. The biology of the shore crab Carcinus maenas (L.). Field Studies Council.

Cuthill, I. C., M. Stevens, A. M. M. Windsor, and H. J. Walker. 2006. The effects of pattern symmetry on detection of disruptive and background-matching coloration. Behavioral Ecology 17:828–832.

Govardovskii, V. I., N. Fyhrquist, T. Reuter, D. G. Kuzmin, and K. Donner. 2000. In search of the visual pigment template. Visual Neuroscience 17:509–528.

Hanlon, R. ., C.-C. Chiao, L. . Mathger, A. Barbosa, K. . Buresch, and C. Chubb. 2009. Cephalopod dynamic camouflage: bridging the continuum between background matching and disruptive coloration. Philosophical Transactions of the Royal Society B: Biological Sciences 364:429–437.

Komdeur, J., M. Oorebeek, T. Van Overveld, and I. C. Cuthill. 2005. Mutual ornamentation, age, and reproductive performance in the European starling. Behavioral Ecology 16:805–817.

Nokelainen, O., N. Hubbard, A. E. Lown, L. E. Wood, and M. Stevens. 2017. Through predators ' eyes : phenotype – environment associations in shore crab coloration at different spatial scales. Biological Journal of the Linnean Society:1–14.

Pike, T. W. 2011. Using digital cameras to investigate animal colouration: Estimating sensor sensitivity functions. Behavioral Ecology and Sociobiology 65:849–858.

Shand, J., J. C. Partridge, S. N. Acher, G. W. Potts, and J. N. Lythgoe. 1988. Spectral absorbance changes in the violet/blue sensitive cones of the juvenile pollack, Pollachius pollachius. Journal Of Comparative Physiology A:699–703.

Siddiqi, A., T. W. Cronin, E. R. Loew, M. Vorobyev, and K. Summers. 2004. Interspecific and

intraspecific views of color signals in the strawberry poison frog Dendrobates pumilio. The Journal of experimental biology 207:2471–85.

Spottiswoode, C. N., and M. Stevens. 2011. How to evade a coevolving brood parasite: egg discrimination versus egg variability as host defences. Proceedings of the Royal Society B: Biological Sciences 278:3566–3573.

Stevens, M., A. E. Lown, and L. E. Wood. 2014a. Color change and camouflage in juvenile shore crabs Carcinus maenas. Frontiers in Ecology and Evolution 2:1–14.

Stevens, M., A. E. Lown, and L. E. Wood. 2014b. Camouflage and Individual Variation in Shore Crabs (Carcinus maenas) from Different Habitats. PloS ONE 9:e115586.

Stevens, M., C. A. Párraga, I. C. Cuthill, J. C. Partridge, and T. S. Troscianko. 2007. Using digital photography to study animal coloration. Biological Journal of the Linnean Society 90:211–237.

Stevens, M., M. C. Stoddard, and J. P. Higham. 2009. Studying primate color: Towards visual system-dependent methods. International Journal of Primatology 30:893–917.

Stoddard, M. C., and M. Stevens. 2010. Pattern mimicry of host eggs by the common cuckoo, as seen through a bird’s eye. Proceedings of the Royal Society B: Biological Sciences 277:1387–1393.

Troscianko, J., and M. Stevens. 2015. Image Calibration and Analysis Toolbox - a free software suite for objectively measuring reflectance, colour and pattern. Methods in Ecology and Evolution 6:1320–1331.

Vorobyev, M., D. Osorio,  a T. Bennett, N. J. Marshall, and I. C. Cuthill. 1998. Tetrachromacy, oil droplets and bird plumage colours. Journal of Comparative Physiology 183:621–33.
